# Treatment of Isolated Posterolateral Tibial Plateau Fracture with a Horizontal Belt Plate through the Anterolateral Supra-Fibular-Head Approach

**DOI:** 10.1155/2020/4186712

**Published:** 2020-12-03

**Authors:** Sunjun Hu, Shiyi Chen, Shimin Chang, Wenfeng Xiong, Rujan Tuladhar

**Affiliations:** Department of Orthopaedic Surgery, Yangpu Hospital, Tongji University School of Medicine, 450 Tengyue Road, Shanghai 200090, China

## Abstract

The posterolateral tibial plateau fracture was not easy to be exposed and fixed with usual techniques. The aim of this study was to investigate the biomechanical stability and clinical outcome of the isolated posterolateral tibial plateau fracture fixed with a single horizontal belt plate through the anterolateral supra-fibular-head approach. Fracture models were created by 18 synthetic tibias and fixed with three different fixation modes. Each group was fixed and tested on the loading machine, and final vertical displacement of the fragment was detected and calculated. Clinically, a retrospective analysis of 12 cases of posterolateral tibial plateau fracture from January 2013 to December 2017 was performed. There were 8 males and 4 females, aged 33-72 years, with an average age of 49.6 years. Isolated posterolateral tibial plateau fractures were identified according to preoperative X-ray and computed tomography scan. Through the modified anterolateral supra-fibular-head approach, the fracture was reduced and fixed by a prebending T-shaped distal radius plate and rafting screws, with bone substitute grafting or autogenous iliac bone implantation. Patients were followed up to a minimum one year of time period, and the outcome was evaluated clinically and radiologically. The biomechanical study shows that horizontal belt plate fixation for the isolated PL tibial plateau fracture can provide sufficient stability, allowing early knee functional exercise and partial weight bearing. For clinical case series, the average operation time in this group was 73.3 ± 10.2 mins (range: 55-90), and the average duration of hospitalization was 9.1 ± 3.3 days (range: 5-16). Patients were followed up for 12-24 months with an average of 16.5 months, and all patients achieved radiological fracture union after an average of 13.7 weeks. At one year after operation, the average knee score of the Hospital for Special Surgery (HSS) scale was 93.2 ± 4.2 points(range: 90-98), the average score of SMFA was 21.1 ± 5.6 points (range: 14-31), and the average knee range of motion (ROM) was 121.48° ± 8.88° (range: 105°-135°). There were 8 cases that were very satisfied and 3 cases that were satisfied with the operation. For an isolated posterolateral tibial plateau fracture, the supra-fibular-head approach can fully expose the fracture site; the horizontal belt plate fixation of the fracture is stable and reliable to allow for early-stage knee rehabilitation, and the outcome of medium-term clinical follow-up was satisfactory.

## 1. Introduction

A posterolateral fracture fragment (PLF) in tibial plateau fractures, either isolated or combined with other tibial plateau quadrants, is not uncommon and often necessitates surgical treatment [1–5]. Isolated PL tibial plateau fractures are relatively less common, with an incidence ranging from 7 to 14% [6, 7]. Although these injuries can be easily confirmed by computed tomography (CT) clinically, it is not easy to expose and fix the fracture using conventional methods, as the main parts of the fragments are usually covered by the fibular head and posterolateral corner structure (PLC), and several neurovascular bundles run across the popliteal cavity, which may interfere with exposure via a posterior incision. Postoperative CT scan also confirmed that the most malreductions were located in the posterolateral quadrant of the lateral plateau [8].

Various approaches that have been described in the literature to expose and fix the PLF can be grossly divided into two classes, anterior approaches and posterior approaches [1, 4, 9–15]. There are three major posterior approaches that are clinically used: (1) the PL approach, through the outer side of the lateral head of the gastrocnemius muscle and soleus, including osteotomy and nonosteotomy approaches, (2) the posteromedial approach, through the inner side of the medial head of the gastrocnemius muscle and soleus, and (3) the posterocentral approach, through the medial and lateral head of the gastrocnemius muscle in the popliteal cavity, which requires the anatomical separation of bundles of blood vessels and nerves.

Traditional anterolateral (AL) approaches are less commonly used for treating isolated PL fractures, according to the literature. As direct visualization of the PLF through a conventional AL incision is often inadequate, several authors have reported the use of modified AL approaches to treat PL tibial plateau fractures [14, 16–18]. Hu et al. [17] reported a modified AL approach (supra-fibular-head approach, SFH). The incision started from Gerdy's tubercle, obliquely upward, across the fibular head, and about 2 cm above the articular line, and fibular collateral ligament (LCL) was dissected, in which the interspace between the FCL and lateral margin of the lateral condyle was used, providing adequate visualization of the PLF and adequate space for placing a narrow plate. Maximal lateral tibial plateau exposure may be obtained with the knee positioned at 110 degrees flexion with an anterolateral rotatory varus force [19].

Currently, there are no implants specifically designed for use in treating PL tibial plateau fractures, and existing lateral anatomical plates may not be placed posteriorly enough to support the PL joint surface fragment with raft screws. Using six different plates from 5 leading manufacturers, a study by Sassoon et al. [18] found that 42% of the entire anteroposterior (AP) depth of the lateral plateau was unsupported and located behind the most posterior raft screw. The average articular surface area that remained unsupported was 40% (range: 25–58%).

Instead of conventional lateral anatomical plates, which are large and thick, we present an available technique to reduce and fix the PLF using a horizontal belt plate (prebent from a 3.5 mm T-shape plate for distal radius fracture) through the AL SFH approach, which can provide simple and effective exposure with rigid raft support for the depressed PL articular surface. The short- to medium-term outcomes in these cases were satisfactory. In this paper, we present our fundamental biomechanical research and experience with the SFH approach and horizontal belt plate fixation in a lateral decubitus position with a single prep and drape process.

## 2. Materials and Methods

### 2.1. Biomechanical Considerations

**Figure 1:**
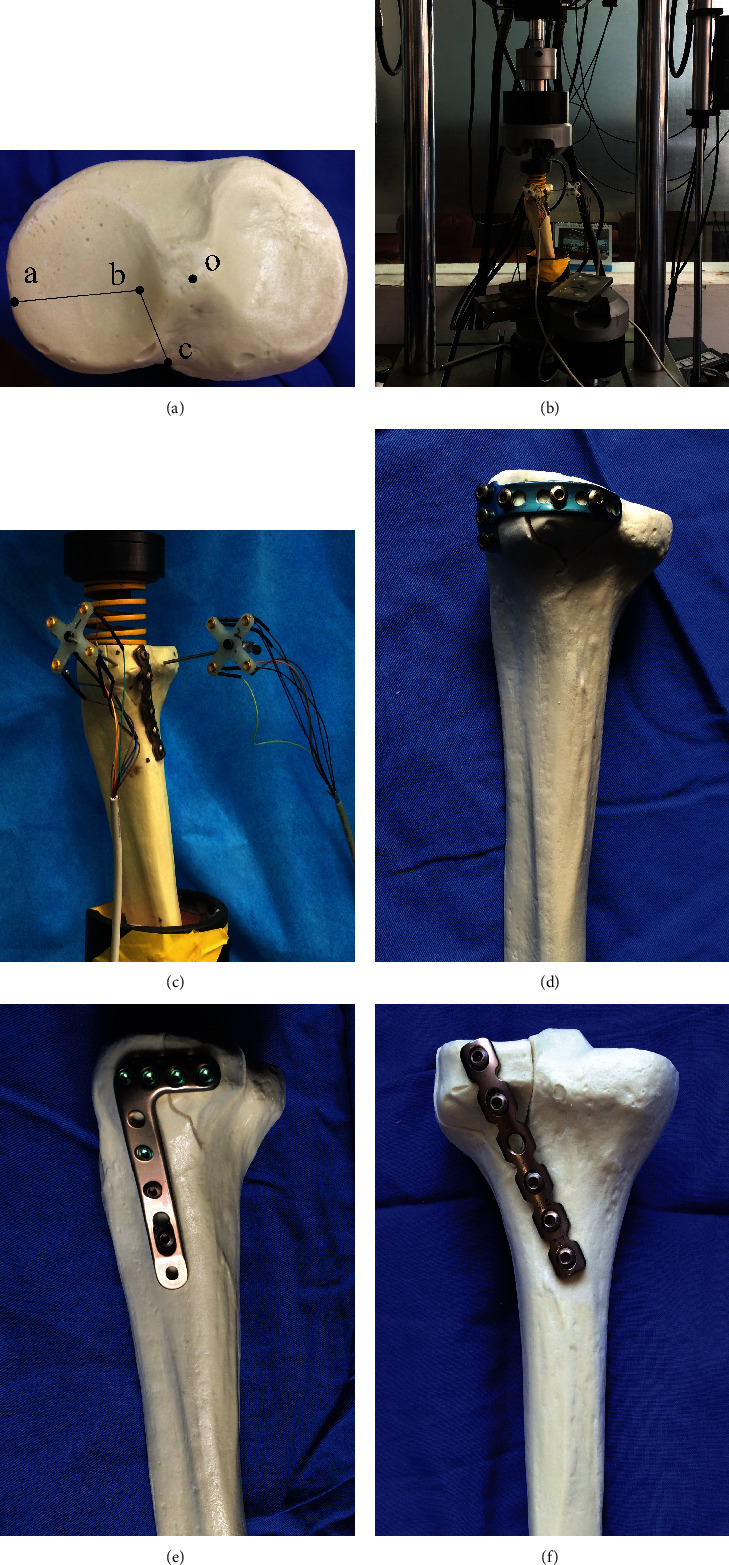
(a) Posterolateral shearing tibial plateau fracture model. Mark a is the lateral edge of the tibial plateau, mark o is the centre of the tibial intercondylar eminence, mark b is the lateral border of the tibial intercondylar eminence, and mark c is the intersection point of the lateral and medial tibial condyles. (b) The specimen was fixed on the testing machine. (c) The mark point of the fracture fragment and the proximal tibia, the connecting wire, and the 3D motion capture system. The posterolateral fracture models of the tibial plateau were randomly divided into 3 groups: (d) horizontal plate, (e) lateral low-profile anatomical locking compression plate, and (f) posterior reconstructive plate.

PL shearing fracture models were created in 18 synthetic tibia (Synbone, type 1110. Synbone AG, Swiss) and randomly assigned to 3 groups. Based on the published literature and clinical morphology data from our department [20], the PL part of the synthetic tibiae was sawed off to simulate a PL shearing fracture. The PLF was fixed using horizontal belt plates (described in this paper, Double Medical Ltd., Xiamen, China) (group A), lateral lower-profile anatomical locking compression plates (3.5 mm LCPs, Synthes, Inc., Oberderf, Switzerland) (group B), and 3.5 mm posterior reconstructive plates (Synthes, Inc., Oberderf, Switzerland) (group C). Loading tests were carried out using a universal hydraulic material tester (The Shore Western Model 107-160 WhisperPak, Shore Western Manufacturing, Inc., USA), and displacement of the specimen was monitored and acquired using the NDI Optotrak three-dimensional motion capture system (Optotrak Certus® motion capture system, Canada) (►Figure 1).

The vertical displacement of the PLF was recorded under axial loading, including a static loading test (from 300 N to 1050 N, simulating a 70 kg adult with partial to full weight bearing) and a fatigue loading test (from 0 N to 1050 N at 10 mm/min and 10,000 repeated loading cycles). During the damage loading test, only the final loading force was required.

### 2.2. Patient Series

The study was approved by the Institutional Ethics Committee with the review board approval number of LL-2016-ZRKX-003. From January 2013 to December 2017, twelve patients with an isolated depressed PL tibial plateau fracture were treated with open reduction and internal fixation with the SFH approach and horizontal belt plate. There were eight men and four women, with an average age of 49.6 years (range: 33-72). The left knee was involved in five cases, and the right knee was involved in seven cases. According to the preoperative imaging examination, 5 cases were classified as Schatzker type III (central depression, articular step-off was more than 10 mm), and 7 cases were classified as Schatzker type II (PL split depression, articular step-off was more than 10 mm). There were no cases with associated neurovascular injury or cruciate ligament rupture found at the time of admission.

### 2.3. Surgical Technique

The patients underwent surgery in a lateral decubitus position, with the injured limb maintained in a slightly flexed position. After sterilization, preparation, and draping, the tourniquet was inflated. The skin incision made was more posterior and shorter than traditional AL incisions, just above the fibular head. An 8 cm long skin incision was made, starting 2 cm below Gerdy's tubercle and extending backwards and upwards, crossing over the fibular head to the joint line. After dissection of the superficial layer, several structures were identified: the iliotibial tract (ITT), the biceps femoris tendon (superficial), and the FCL (deeper). The ITT was cut along the direction of the fibers, and the posterior part of the tendon termination on Gerdy's tubercle was dissected for exposure, while the space between the FCL and lateral condyle was developed. The inferior margin of the coronal ligament and joint capsule was cut open, and sutures in the lateral meniscus were used for traction of the meniscus. After clearing the haematoma in the articular cavity, two meniscus hooks were used for exposure, the FCL was mobilized for posterior retraction with the knee internally rotated and flexed, and the PL articular surface was clearly visualized.

A cortical window was developed on the AL metaphysis approximately 2 cm below the articular surface. The depressed articular fragment was elevated using an osteotome to restore the congruence of the articular surface. A K-wire 1.5 mm in diameter was used to maintain the reduction. Alternative bone void fillers can be used to fill metaphyseal defects after elevation, as well as iliac crest autografts and allogeneic bone grafts.

A 3.5 mm T-shaped plate designed for distal radial fractures or posterior malleolar fractures was used for PLF enclosure and fixation. One arm of the plate was cut off, and the plate was prebent to fit the margin of the lateral condyle. The plate was placed horizontally below the subchondral bone with the end of the plate placed on the interspace above the fibular head. For a split and collapsed fracture with a significantly posteriorly displaced fragment (Schatzker type II), a longer plate was chosen, and the end of the plate was prebent and inserted into the backside of the PL condyle. For a simple depressed PL fracture (Schatzker type III), a plate with 4 holes in length was sufficient for fixation. Typically, at least 2 screws can cross over the PLF, and the screws were long enough to bridge the medial condyle and stabilize the fragment.

After irrigating the incision, the opened coronary ligament and the released iliotibial band fiber were sutured back before incision closure. No drains were used in this group.

### 2.4. Postoperative Management

A plaster cast was not used to immobilize the knee joint. The lower limbs were elevated on a trapezoidal cushion to promote detumescence. Patients were instructed to undertake quadriceps strengthening exercises and practice straight leg raise exercises immediately. Continuous passive motion was applied using a machine or by an assistant to allow less than 60° knee flexion in the first week and was increased gradually to 90° in the second week. Patients were encouraged to gain full range of motion (ROM) in four weeks. Weight bearing was restricted to toe touch (5-10 kg) during the first month and increased progressively during the second month. At 8 weeks after the operation, the brace was removed, and the patients were encouraged to progress to full weight bearing.

The patients were followed up at 6 weeks, 3 months, 6 months, 1 year, and 2 years postoperatively, with clinical and radiographic assessments of the progress of healing and complications. The motion of the knee joint was assessed by physical examination. Postoperative CT scans and follow-up radiographs were taken during the follow-up evaluations. The knee ROM was measured, and functional outcomes were assessed by the Hospital for Special Surgery (HSS) knee scoring system and Short Musculoskeletal Function Assessment (SMFA) score at the final follow-up visit.

## 3. Results

The biomechanical results are summarized in Table 1. The average displacement in groups A, B, and C was 1.23 ± 0.23 mm, 1.17 ± 0.22 mm, and 0.91 ± 0.26 mm, respectively, under a loading force of 1050 N, and there were no significant differences among the groups (*P* < 0.05). The average displacement in groups A, B, and C was 2.77 ± 1.79 mm, 2.69 ± 1.14 mm, and 1.62 ± 0.60 mm, respectively, under fatigue loading; there were no significant differences among the groups (*P* > 0.05). Under damage loading, the final force in groups A, B, and C was 2055 ± 263 N, 1968 ± 209 N, and 2272 ± 130 N, respectively, and there was a significant difference among the groups (*P* < 0.05). This study demonstrates that the horizontal belt plate fixation for the isolated PL tibial plateau fracture can provide sufficient stability for the PLF. Although the definite support and strength of the posterior plate fixation is better than those of the other two fixation methods, the horizontal belt plate fixation may also allow early knee functional exercise and partial weight bearing.

**Figure 2:**
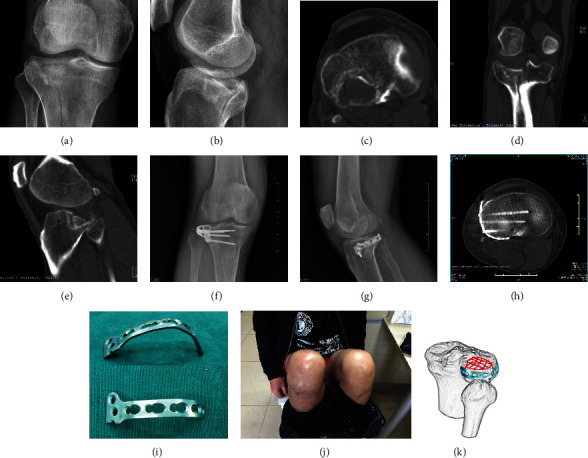
Typical case: a 50-year-old male fell from a height. (a)–(e) Preoperative X-ray films and CT images confirmed posterolateral quadrant tibial plateau fracture. (i) Application of the horizontal belt plate on the posterolateral tibial plateau: a 3.5 mm T-shaped plate was used; one arm of the plate was cut off, and the plate was prebent to fit the margin of the lateral condyle (i). (f)–(h) Postoperative X-ray films and axial CT images showed satisfactory reduction of the posterolateral fracture and congruence of the articular surface. (j) The knee function was satisfactory at the final follow-up visit. (k) 3D schematic diagram of the postoperative belt plate fixation mode.

The average operative duration in this group was 73.3 ± 10.2 min (range: 55-90), and the average hospitalization duration was 9.1 ± 3.3 days (range: 5-16). All incisions healed with no cases of deep infection, vascular injury, peroneal nerve paraesthesia, or compartment syndrome. All fractures were healed, as manifested by painless weight bearing without a brace and by radiographic assessment. No signs of lateral or PL instability of the knee were demonstrated during the follow-up period. Over an average follow-up period of 16.5 months (range: 12-24), all patients achieved radiological fracture union after an average of 13.7 weeks and returned to their preinjury work. According to the final assessment, the average knee ROM was 121.48° ± 8.88° (range: 105-135), the average HSS score was 93.2 ± 4.2 (range: 90-98) points, and the average SMFA dysfunction score was 21.1 ± 5.6 (range: 14-31) points. The information of a typical case demonstrates good function (►Figure 2).

## 4. Discussion

PL tibial plateau fractures usually result from axial loading with the knee in a flexed or semiflexed position, and the tibia has a tendency towards anterior subluxation on the femur, which may be hit by the lateral femoral condyle [21]. The wide use of low-speed vehicles (less than 40 km/h) may cause a relatively high incidence of these lesions [22]. There are two major morphological types of isolated PL tibial plateau fractures according to radiological images: simple depressed fractures with a relatively intact posterior cortical wall and depressed and split fractures with a ruptured posterior cortical wall and displaced posterior cortical fragment. These fractures may be refined as type III or type II by the Schatzker classification system or AO/OTA 41B1.1 (4), 41.B2.2 (4), or 41.B3.1 (2) according to the AO classification system. [23] Although the PL fracture is not as common as the lateral or medial condylar fracture, a retrospective radiographic review by Xiang et al. [7] confirmed that the average magnitude of PLF displacement was 10.5 ± 5.2 mm (range: 2–19 mm), and that the average maximum posterior cortical height was 29 mm (range: 18–42 mm); such fractures may require a surgical intervention to restore the congruence of the articular surface. Collapse of the PLF may increase the risk of posttraumatic arthritis or lateral knee instability in the long-term.

### 4.1. Approaches for PLF

Several alternative surgical approaches have been reported in the literature for direct PLF exposure and plate fixation with good short-term outcomes [1, 8–12]. As it is covered by the fibular head and ligamentous structures, it is difficult to reduce the PLF through a traditional AL approach; posterior approaches are instead prevalent and favoured by authors. The PL approach (osteotomy-free) described by Carlson et al. [15] and modified by Chang et al. [10] has the merit of allowing direct visualization and rigid posterior supporting plate fixation. However, the anterior tibial vascular bundle passes through the superior fissure of the interosseous membrane to the anterior compartment, which may restrict lengthening of the incision or cause iatrogenic vessel rupture [24–26]. The PL approach reported by Frosch et al. [14] was performed in the lateral decubitus position to expose both the AL and PL quadrants, which required extensive soft tissue dissection and is therefore not suitable for use in treating unicondylar PL fractures. The PL transfibular approach (needs osteotomy) described by Lobenhoffer et al. [1] and Solomon et al. [4] carries the risk of nonunion or malunion at the osteotomy site. All these approaches require intraoperative dissociation of the common peroneal nerve (CPN), which not only increases the operative duration but also increases the risk of injury to the CPN.

As the traditional AL approach cannot provide full visualization of the posterior articular surface or utilize the buttress plate, several authors have reported new methods to improve visualization. A lateral femoral epicondylar osteotomy and a submeniscal approach were introduced by Yoon et al. [27] for increased intraoperative exposure, while a partial fibular head osteotomy reported by Yu at al [16] was used to expose and manipulate the PL fracture. We also reported plating PL fractures through a modified AL SFH approach [14]. According to our study, the interspace between the apex of the fibular head and the lateral condylar surface allows direct visualization of the PL articular surface and the possibility of placing the plate more posteriorly.

Compared with other approaches, the SFH approach has the following advantages: (1) it is a simple and easy AL approach not involving any vital neurovascular structures. (2) It avoids the covering of the fibular head to the PL platform, and the articular surface can be reduced under direct vision. (3) The lateral L-shaped locking plate can be placed posteriorly above the slope of the fibular head, and the most posterior screw can be placed at the insertion of the FCL, thus reliably supporting the posterior articular surface. (4) By inserting the prebent horizontal belt plate into the backspace of the lateral condyle through the SFH space, it can wrap up the PL cortex of the lateral condyle and form a hoop plate for the PLF. (5) It can be used for treating isolated PL fractures, combined PL and AL fractures, and dual condylar fractures involving the PL quadrant.

As for the difficulty of reconstructing the articular surface of comminuted tibial plateau fractures, using an arthroscope through the open surgical approach for visualization of the fracture line and evaluation of fracture reduction in complex tibia plateau fractures has been reported [28, 29]. Krause et al. [29] recommended for tibial plateau fractures involving the postero-latero-central region of the lateral tibial condylar and intraoperative arthroscopy permitted a remarkable improved visualization of fracture reduction.

### 4.2. Horizontal Belt Plate

As current plates may not be suitable for posterior positioning with raft screws to support the PL joint surface, supplementary methods were introduced to strengthen the support of the PLF [18, 30, 31], such as the use of an intraosseous fibular shaft allograft as a reduction tool and structural support [18]. Sun et al. [26] also introduced a single screw obliquely implanted from anterior-inferior-medial to posterior-superior-lateral to enhance the fixation stability of the lateral rafting plate. However, the exact position of the screw is difficult to grasp by intraoperative manipulation and fluoroscopy; without the support of the plate as a frame, the definite fixation effect of the point-to-point resistance is limited.

Although the use of the belt plate technique has been introduced by authors, the treatment of isolated PL fractures with a single band plate has not been reported in the literature [30–34]. In 2008, Bermudez et al. [33] reported the use of a 3.5 mm reconstruction plate as a horizontal plate and a metaphyseal supporting plate to treat two cases of high-energy comminuted tibial plateau fracture. In 2016, we reported the treatment of an isolated PL fracture with a horizontal rafting plate bent from a 3.5 mm T plate for the distal radius [17]. Cho et al. [34, 35] reported the use of a 2.7 mm rim plate and lateral LCP for plating a comminuted lateral condyle fracture via a modified AL approach. Giordano et al. [32] introduced the “hoop plating” technique, performed by placing a horizontal, precontoured 1/3 tubular plate wrapped around the posterior rim of the tibial plateau. The implant was placed exactly on the posterior cortex of the tibial plateau and could provide containment for the reduced juxta-articular posterior cortex and rim.

There are several merits of the T-shaped horizontal rafting plate used in this work. (1) Unlike a reconstruction plate, it offers three-dimensional fixation with screws in the horizontal and sagittal planes (lateral column). The fixation force is stronger than that of a single straight plate. Our biomechanical study confirmed that the horizontal belt plate fixation can achieve PL fragment stability equivalent to that of a standard LCP or posterior buttress plate. (2) A smaller incision can be used compared with that needed for a standard proximal tibial LCP, with less tissue dissection, and patients can recover faster after the operation. (3) The plate trunk is relatively thin and can be inserted to the back of the lateral condyle (via the SFH space) to wrap around the entire external condylar rim. (4) A 3.5 mm T plate is less expensive than a standard LCP, thus reducing the financial burden on patients.

As none of the patients in this group suffered from a severe high-energy injury, a single horizontal belt plate may be insufficient for all lateral condylar depressed fractures with epiphyseal comminution, which may need an additional strong LCP. [36]

## 5. Conclusions

Although there are still some deficiencies in this study, the novel technique we have introduced is easy and effective. For the treatment of isolated PL quadrant tibial plateau fractures, the modified AL SFH approach can easily and fully expose the fracture site and provide stable and reliable fracture fixation with a horizontal belt plate, allowing early-stage knee rehabilitation. In this study, the mid-term outcomes of this approach were satisfactory.

## Figures and Tables

**Table 1:** The vertical displacement of the posterolateral fragment fixed by different internal plates under different axial loads (*X* ± *s*, *n* = 9).

Group	Static loading test (mm)			Fatigue test (mm)	Failure load (N)
	350 N	700 N	1050 N		
Group A	0.24 ± 0.07	0.71 ± 0.02	1.23 ± 0.23	2.77 ± 1.79	2055 ± 263
Group B	0.25 ± 0.03	0.70 ± 0.04	1.17 ± 0.22	2.69 ± 1.14	1968 ± 209
Group C	0.22 ± 0.01	0.69 ± 0.03	0.91 ± 0.26	1.62 ± 0.60	2272 ± 130

*F* value	0.982	0.903	4.231	2.287	5.102

*P* value	0.389	0.419	0.027	0.123	0.014

## Data Availability

The data used to support the findings of this study are available from the corresponding author upon request.
